# How adults’ experience with words changes over time: Insights from five years of the Wesleyan Word Experience Project

**DOI:** 10.3758/s13428-025-02765-5

**Published:** 2025-07-28

**Authors:** Barbara J. Juhasz, Grace Devanny, Abby Frankenberg, Ava Galdenzi, Lumen Constance Hirwa, Wiralpach Nawabutsitthirat, Meiwen Shao

**Affiliations:** Department of Psychology, Wesleyan University, Middletown, CT 06459 USA

**Keywords:** Familiarity, Age-of-Acquisition, Word recognition, Lexical change

## Abstract

This study explored how adults’ experiences with words may change over time. Ratings of age-of-acquisition (AoA) and familiarity were collected on 499 words from introductory psychology students each semester, for a total of 5 years. Over the course of ten semesters, ratings were collected from more than 1000 students. One goal of this multi-year project was to explore whether it is possible to assess lexical change for particular words. Regression analyses were used to track the trajectory of the words. Based on the analyses, words were categorized into three different groups: words that remain consistent in AoA and familiarity, those with upward trajectories, and those with downward trajectories. A total of 63 words were identified as undergoing experience-based lexical change. The project also explored how AoA and familiarity relate to each other over time as well as each variable’s interrater reliability over the course of 5 years. Both AoA and familiarity had strong correlations when comparing ratings collected from the first five semesters of the project with ratings collected from the last five semesters of the project. These correlations were significantly higher than the correlation between the two variables, supporting the idea that they are separate constructs. Average familiarity and AoA for the entire set of words did not differ as a function of time. In addition, AoA was found to have the highest correlation with itself, providing evidence that college undergraduates consistently apply the rating scale when asked to assess the age at which words are first learned.

## Introduction

The purpose of this study is to introduce the Wesleyan Word Experience Project, which tracked individuals’ subjective experiences with words over a period of 5 years in an undergraduate student community. Experience with words is necessary to support automatic word recognition during skilled reading. According to Perfetti’s (2017) Lexical Quality Hypothesis (LQH), identifying a written word consists of the concurrent activation of the word’s orthographic, phonological, and semantic representations. Words that have high lexical quality in the mental lexicon have well-specified representations and strong binding between the levels of representation. Experience with reading should lead to higher average lexical quality overall in a reader’s mental lexicon. Thus, individuals can differ in their average lexical quality based on their past reading experience. However, the mental lexicon contains words that vary in lexical quality even for highly skilled readers. Words with high lexical quality can be identified automatically, leaving cognitive resources available for higher-level processes such as reading fluency and comprehension. As noted by Nation (2017), when children experience words in diverse language contexts, this creates a “lexical legacy” that facilitates the development of reading expertise. Therefore, experiencing words in diverse contexts supports the development of accurate representations as well as strengthens the connections between these levels of representation, supporting word recognition and reading comprehension.

Word frequency, or how often a word is encountered in text or speech, provides one way to measure lexical experience. The word frequency effect is ubiquitous in the word recognition literature (see Brysbaert et al., 2018 for a recent review). Words that occur with a high frequency in a given language are processed faster than words that occur with a low frequency across a wide range of word recognition and reading tasks. Word frequency is often considered an objective measure of word exposure, as it is based on counts of how often words occur in a corpus of language. However, not all word frequency measures are equally effective at predicting word recognition time (e.g. Brysbaert et al., 2018, Brysbaert & New, 2009). Issues related to the age of the corpus, the sources that the corpus was developed from, and the size of the corpus can all impact the effectiveness of the word frequency variable at measuring actual exposure with a given word for an individual reader. It is important to keep in mind that an undergraduate student in 2024 may not have experienced words at the same rate as indicated by a frequency norm developed from written sources in the 1960s.

In addition, there are many words that are objectively low in frequency that are still recognized quickly and accurately (Brysbaert et al., 2018; Gernsbacher, 1984). Thus, while frequency is usually the strongest predictor of word recognition time, it is definitely not the ONLY variable that influences processing. Gernsbacher (1984) introduced the idea of “experiential familiarity”, as a subjective rating of “*how often a subject has encountered a word*” (page 277). Gernsbacher noted that inconsistent results in past word recognition studies could be due to differences in experiential familiarity between the high- and low-frequency print words used in previous studies. When experiential familiarity was rated by undergraduate students on a 1–7 scale and used in place of printed frequency measures, Gernsbacher (1984) observed strong main effects of familiarity across multiple experiments. In addition, interactions with other variables (e.g., bigram frequency), that had previously been reported in the literature were no longer significant when experiential familiarity was used in the analysis in place of word frequency.

Experiential familiarity with words can thus be used as a proxy for printed frequency to measure exposure to words. It can also be used in concert with word frequency to explain additional variance in word processing that is not captured by objective measures of print. Experiential familiarity is a subjectively rated variable that has been operationalized in two different ways. In some studies, participants are asked to rate the *subjective frequency* of certain words using instructions that stress how often they have personally encountered a particular word (Balota et al., 2004; Brysbaert & Cortese, 2011). In other studies, participants are asked to rate words more broadly on how familiar they seem overall (Gernsbacher, 1984; Juhasz et al., 2015; Scott et al., 2019). It has been suggested that instructions that call for a judgement of overall familiarity may encourage participants to recruit semantic knowledge about the word in making their judgements (e.g., Brysbaert & Cortese, 2011) relative to instructions stressing encounters with the words.

Research has shown that familiarity/subjective frequency can impact word processing over and above printed word frequency in a number of tasks including word naming (Balota et al., 2004; Cortese & Khanna, 2007; Juhasz et al., 2015), lexical decision (Balota et al., 2004; Cortese & Khanna, 2007; Juhasz et al., 2015), and reading while eye movements are recorded (Juhasz & Rayner, 2003; Juhasz, 2018; Williams & Morris, 2004). In each of these tasks, words with high-rated familiarity were processed faster than words with low-rated familiarity. In fact, Williams and Morris (2004) showed that word frequency no longer influenced fixation durations on target words when rated familiarity was controlled. Conversely, there were significant effects of rated familiarity on fixation durations when word frequency was controlled. However, there has been debate about whether subjective frequency still affects word recognition when more valid measures of objective word frequency are used in predictive models. Brysbaert and Cortese (2011) observed reduced effects of subjective frequency on lexical decision and word naming of monosyllabic words when included in models with more effective measures of word frequency. This stands in contrast to the findings of Juhasz et al. (2015) who continued to observe effects of rated familiarity on compound word processing when included in models with effective measures of printed frequency. This may indicate that experiential familiarity is particularly important for explaining variance in the recognition of morphologically complex words, which tend to be low in printed frequency.

Another experience-based variable that has been discussed in the literature is age-of-acquisition (AoA). AoA is a measure of when a word is first learned by an individual. In most cases, AoA is subjectively rated by adults, although more objective measures of word learning age also exist (e.g., Brysbaert, 2016; Morrison et al., 1997). When AoA is rated, it is often using the 1 to 7 scale first developed by Gilhooly and Logie (1980) where the values of 1–6 on the scale represent a 2-year age band and 7 indicates a word that was learned past age 13 (e.g., Cortese & Khanna, 2008; Juhasz et al., 2015; Schock et al., 2012; Scott et al., 2019). Alternatively, Kuperman et al. (2012) asked adults to provide the age at which they think they learned approximately 30,000 English words. Obviously, adults do not possess high-fidelity autobiographical knowledge of when they first learned each word contained in their mental lexicon. This has led to critiques regarding AoA ratings and whether they actually represent the age of acquisition for words (see Brysbaert, 2016 for a discussion). For example, adults rate words as being later acquired, on average, relative to estimates of when words are first used in corpora of child speech (e.g., Smolík & Filip, 2022). In addition, it has been argued that college students, who are often the individuals who rate AoA in word recognition studies, do not have enough experience with young children to provide valid ratings (Wikse Barrow et al., 2019). In contrast, Brysbaert (2016) demonstrated that adult-rated AoA had the best criterion validity when compared to other measurements of word learning age, such as frequency trajectory and AoA ratings corrected for word frequency. Moreover, rated AoA has also been found to have high interrater reliability in several studies (e.g., Cortese & Khanna, 2008; Juhasz et al., 2015; Schock et al., 2012). Thus, past research converges in supporting the validity and reliability of AoA ratings completed by adults.

There are similarities between the research literature exploring familiarity and AoA. AoA has also been found to affect word recognition in a variety of tasks including word naming, lexical decisions and eye movements in reading (see Elsherif et al., 2023 for a recent review of AoA research). In addition, there has also been debate as to whether AoA is simply another way to subjectively measure the frequency of exposure. Zevin and Seidenberg (2002) noted that previous studies observing effects of AoA using a factorial approach did not adequately control their early and late AoA conditions on measures of printed frequency from up-to-date corpora. As such, they argued that apparent AoA effects in those studies could be attributable to word frequency instead. However, Brysbaert and Cortese (2011) have shown that AoA still accounts for a significant proportion of variance in word naming and lexical decision even when more effective measures of printed word frequency are included in the same models.

Subjective ratings of AoA and familiarity are also clearly related to each other, as they both indicate an individual’s personal experience with words. Juhasz et al. (2015) localized both variables at what they called the lexical/semantic level of processing. As both ratings measure personal experience with words over time, they may impact access to all levels of representation in the mental lexicon. A word that is learned early in life and is highly familiar will speed up access to its lexical representations (orthographic and/or phonological) as well as its intended meaning. Thus, early acquired words that are highly familiar should demonstrate a high degree of lexical quality. On the other hand, a word that is learned later in life and has a low degree of familiarity should be slower to recognize, perhaps due to underspecified lexical and semantic representations. These words thus demonstrate a low level of lexical quality in the mental lexicon. In support of this relationship between the variables, many studies that include both variables report a strong correlation between them. To illustrate this, Table 1 includes the reported correlations between rated AoA and familiarity/subjective frequency for a sample of seven studies using English stimuli. With the exception of Juhasz and Rayner (2003), which will be specifically discussed below, the correlations are all in the strong range, according to Cohen’s (1988) guidelines, with an average correlation of *r* = – 0.689. Thus, words that are learned earlier in life tend to be higher in ratings of familiarity on average.

**Table 1:** Correlations between AoA and familiarity in a sample of published studies

	Correlation	Number of items
Brysbaert & Cortese (2011)	–.722	2336
Cortese & Khanna (2007)	–.721	2342
Elsherif et al. (2020)	–.65	226
Juhasz & Rayner (2003)	–.181	72
Juhasz (2018)	–.70	120
Juhasz et al. (2015)	–.67	629
Scott et al. (2019)	–.67	5553

*Note*. Brysbaert and Cortese (2011) and Cortese and Khanna (2007) used ratings of subjective frequency. Most items overlapped in these two studies. The remaining studies used ratings of overall familiarity. Elsherif et al. (2020) and Juhasz (2018) used a subset of items from the ratings collected by Juhasz et al. (2015)

The relationship between AoA and familiarity has also been explored through factor analysis. Scott et al. (2019) collected ratings for 5500 words as part of their Glasgow norms. Using factor analysis with nine psycholinguistic variables, they found that AoA and familiarity loaded on the same factor, which they labeled “exposure”. Bates et al. (2001) also conducted factor analysis to explore word and picture naming in Italian. Familiarity and subjective AoA loaded on two factors in this study. The first factor also included measures of word frequency, thereby again representing word exposure. The second factor included AoA, familiarity, and the semantic variables of imageability and concreteness. Bates et al. note that the second factor loading makes sense theoretically, as children’s first words tend to be names of familiar, concrete objects. They further suggest that word familiarity ratings may represent a measure of subjective frequency as well as conceptual accessibility. Taken together, these factor analyses further support the relationship between AoA and familiarity as measures of word exposure. Bates et al. (2001) also provide support for the idea that these variables may impact access to lexical and semantic representations in the mental lexicon.

While AoA and familiarity are both subjective measures of experience with words, it is important to note that they can also be dissociated from each other. As indicated in Table 1, Juhasz and Rayner (2003) selected 72 words for a reading study with a specific goal to minimize the correlations between predictor variables in order to reduce multicollinearity in their regression analyses. In this study, readers’ eye movements were recorded while they read sentences containing the target words. Familiarity had an early and long-lasting effect on fixation durations on the target word. It influenced all reading time measures that were explored when included in a regression model with word frequency, length, AoA, and concreteness. AoA was also found to impact fixation durations on the target words when both frequency and familiarity were controlled, a finding that has been replicated (e.g., Juhasz & Sheridan, 2020). Thus, familiarity and AoA both separately impact the reading of words that are included in sentences.

As mentioned above, frequency norms can be used as a more objective measure of word exposure. However, they represent the frequency with which a word was encountered during one particular time period. Languages change and evolve over time, and objective frequency norms can be time-consuming to calculate and update regularly. Thus, their effectiveness at predicting word recognition will decrease as they age (see Brysbaert & Cortese, 2011 for a discussion). Collecting ratings of AoA and familiarity can be relatively easy and may thus represent a more accurate measure of actual current word experience for a given population of individuals. There are many corpora of AoA and/or familiarity ratings that have been published in the last two decades to aid researchers with their stimuli selection (e.g., Cortese & Khanna, 2008; Juhasz et al., 2015; Kuperman et al., 2012; Schock et al., 2012; Scott et al., 2019). However, it is important to keep in mind that these ratings are also specific to a particular time and geographic region. Thus, a question can be raised about how sensitive AoA and familiarity ratings are to the passing of time.

As discussed by Beckner et al. (2009), languages can be thought of as a complex adaptive system (CAS) that exist at both an individual and societal level. Languages thus undergo usage-based changes over time due to individual variations in the replication of language forms and diversity of usage across speakers of that language. At the lexical level, change can occur through the addition of new words to the lexicon. According to Simonini (1966), there are 15 ways that new words enter the collective lexicon in the English language, including compounding, derivation, blending and creating acronyms. In addition, lexical change can occur when words change their meaning, for example by using a concrete noun to refer to a more abstract, metaphorical meaning (Bybee, 2015). The role of social interaction is also important to consider with respect to lexical change, as words may be used differently across social groups and age bands. Thus, words can be more common in some geographical regions or with certain affinity groups.

Current events, technological advancements, and social movements can be powerful agents of language change. As one example, let’s consider the word “pandemic”. According to the English Lexicon Project (Balota et al., 2007), “pandemic” is a low-frequency word (0.100 instances per million) that is acquired relatively late (12.26 years). However, these measures of exposure were collected prior to 2020. This word has most likely become much more common over recent years due to the COVID-19 pandemic that impacted individuals across the globe. This unprecedented societal event may have driven lexical change that has most likely affected the age at which words related to the pandemic were acquired as well as their overall familiarity.

How can lexical change be measured? In the case of new words that are introduced into a language, this can be tracked via corpus-based analysis of speech, online discussions, or additions to published dictionaries. As noted in Beckner et al. (2009), it is usually not feasible to explore language use at a community level in a timescale that allows for exploration of language changes and evolution. On the other hand, it may be possible to track more subtle variations in exposure and word usage if measurements of subjective ratings of experience are taken regularly over time within a community. The Wesleyan Word Experience Project (WWEP) was developed to explore potential lexical change by tracking variations in rated familiarity and AoA over 5 years. All participants were undergraduates who chose to take Introductory Psychology at a specific, selective liberal arts college in the northeastern United States, thereby minimizing lexical variation due to geographical region, age, and level of education.

There were four main goals that the WWEP was developed to meet. First, by collecting familiarity and AoA ratings over a period of 5 years, we hoped to explore instances of lexical change over time. Specifically, the trajectories for both familiarity and AoA were assessed for the ten semesters of data collection. Words were categorized into those that remained consistent in their ratings over the course of the 5 years, those that demonstrated an upward trajectory in AoA and familiarity over time, and those that demonstrated a downward trajectory over time. A period of 5 years was selected as the initial goal for this project to explore whether it is possible to use ratings to track subtle lexical change within a generational cohort. We expected to observe words in all three trajectory categories (upward, consistent, downward) due to changes in collective lexical experience by college undergraduates over the time span of 5 years. Specifically, social media usage, changes in technology, and global events should shape the lexical experiences of emerging adults. In addition to our own analyses, the average ratings for these two variables and the number of observations per semester are also available as supplemental material to this article so that interested researchers can conduct their own analyses to explore lexical change.

The second goal of the WWEP was to explore the interrelationship between AoA and familiarity over time. As noted above, AoA and familiarity tend to be highly correlated in the literature. They are both subjectively rated measures of experience with words and load on the same underlying factors in previous studies (e.g., Bates et al., 2001; Scott et al., 2019). This raises the question as to whether AoA and familiarity should be considered separate constructs. One way to answer this question is to explore the correlations between AoA and familiarity over time. We expected the correlation for both AoA and familiarity to be in the range reported in past studies (see Table 1) and that the relationship should remain stable over the course of 5 years.

The third goal of the study was to measure interrater reliability for both variables (AoA and familiarity) over time. In order to accomplish this, we explored how strongly the ratings of AoA from the first five semesters of the project correlated with AoA ratings from the last five semesters. The correlation of ratings for the first and last five semesters was also calculated for the familiarity ratings. The strength of the correlations was then compared to assess whether the rated variables differed from each other in terms of interrater reliability. Based on past research (e.g., Juhasz et al., 2015), we expected both variables to demonstrate strong interrater reliability. We did not have a prediction as to whether the variables would differ in their interrater reliability.

The fourth goal was to assess the continued value of the ELP’s behavioral measures as a window into word recognition for college students in the 2020s. The ELP was first introduced in the literature in 2007 (Balota et al., 2007). It reports word naming and lexical decision data collected from undergraduates at six universities and has been a useful resource for word recognition researchers. While the ELP has been an incredibly rich source of data, the question can be raised as to whether the applicability of its behavioral measures diminishes over time as lexical change occurs. Thus, we compared the correlations between the ELP behavioral results and the ratings of familiarity and AoA over the course of the 5 years of the WWEP. We predicted that the relationship between both variables and the ELP behavioral results would weaken over time.

## Method

### Participants

A total of 1036 participants contributed data towards the WWEP between the Fall 2019 and Spring 2024 semesters. Participants were recruited from the Introductory Psychology course at Wesleyan University and participated in exchange for partial course credit. The number of participants varied each semester due to the size of the Introductory Psychology course(s) and the number of labs recruiting from the participant pool. Participants were assigned to the project as one of their research experience credits for the semester. Each participant was then randomly assigned to one of the four WWEP questionnaires. Demographic information was collected from the participants at the start of each questionnaire. Demographic information is summarized in Table 2. In addition to the participants who contributed data to this project, a small number of participants’ data were not included due to failure to provide consent, not providing any ratings, completing the questionnaire more than once, or completing the online questionnaire in a language other than English. No additional exclusion criteria were applied.

**Table 2:** Demographic characteristics of WWEP participants

Variable	*n*	*%*	*M*	*SD*
Age	1036		18.9	1.17
Gender				
Male	425	41		
Female	611	59		
Class year				
First year	698	67.4		
Sophomore	223	21.5		
Junior	73	7		
Senior	38	3.7		
Did not say	4	.4		
First language				
English	871	84.1		
Other	165	15.9		

*Note*. *n* = number of participants, M = mean, SD = standard deviation

### Materials

The WWEP words were selected from three sources. The largest set of words (383) was selected from a randomized list of words generated from the English Lexicon Project (Balota et al., 2007; e.g., celebrity, vague). Fifty-two words were included from stimuli that were being used in other word recognition projects in the Wesleyan Eye Movement and Reading Lab in 2019 (e.g., vitamin, rattlesnake). Finally, 65 words were generated by undergraduate research assistants as examples of words that may change in their familiarity to typical college students over the next decade (e.g., tweet, gluten). The original word list contained 500 words. However, one word was inadvertently left off the initial questionnaires in Fall 2019, resulting in 499 words that were included on the WWEP. The 499 words were split into two lists (250 and 249). Each participant rated one list on familiarity and one list on rated AoA. Both ratings used a 1–7 scale.

For the familiarity ratings, a higher number indicates greater familiarity with the word. The instructions were the same as those used in past studies by the first author (e.g., Juhasz & Rayner, 2003; Juhasz et al., 2015). The participants were provided with the vertical list of words in a fixed random order and were asked to click on the circle to the right of each word corresponding to the 1–7 scale that represents their familiarity level with the word. The scale was anchored with the words 1 = not familiar, 4 = somewhat familiar, and 7 = very familiar. The intermediary values (2, 3, 5, and 6) were not labeled.

The instructions for the AoA ratings were the same as those used in past studies by the first author (Juhasz & Rayner, 2003; Juhasz et al., 2015) and utilized the 1–7 AoA scale first developed by Gilhooly and Logie (1980). The words were presented in a fixed random order in a vertical list, and participants clicked the circle that represented their estimate of when they first learned each word. Each value on the 1–7 scale was labeled with the corresponding Gilhooly and Logie age category (1 = 0–2 years of age, 2 = 3–4 years of age, 3 = 5–6 years of age, 4 = 7–8 years of age, 5 = 9–10 years of age, 6 = 11–12 years of age, 7 = 13+ years of age). Participants were allowed to skip rating words for both questionnaires.

Four versions of the WWEP were created each semester. Version 1 asked participants to first rate List 1 words on AoA followed by List 2 words on Familiarity. Version 2 reversed the order of questionnaires such that Familiarity ratings for List 2 words were presented first followed by AoA ratings for List 1 words. Version 3 presented List 2 words for AoA ratings first followed by List 1 words for Familiarity ratings. Version 4 presented List 1 words for familiarity ratings first followed by List 2 words for AoA ratings.

### Procedure

At the start of each semester from Fall 2019 to Spring 2024, participants from Introductory Psychology were assigned to the WWEP as one of their research exposure requirements. Once the list of participants was received, they were randomly assigned to one of the four WWEP questionnaires that were administered as a Qualtrics survey. Participants were invited to complete their assigned questionnaire and were given approximately 2–3 weeks to do so, in exchange for course credit. They were told via e-mail that the online questionnaire should take approximately 45 min to 1 h to complete. They were asked to set aside this amount of time to complete the online study and to complete it on a computer in a quiet place. Participants first read and completed an informed consent form. They then completed a brief demographic questionnaire, followed by two sets of word ratings. A debriefing statement ended the online questionnaire.

### Data analysis

At the end of data collection each semester, the data were downloaded from Qualtrics and an initial data processing stage was conducted. In this stage, the three sections of the data file were separated (demographics, AoA and familiarity) and any incomplete/ineligible participant files were deleted. The averages for AoA and familiarity for each item were then computed and added to the master WWEP spreadsheet for analysis. These averages, which are included in the supplemental information for this article, were the basis of the by-item correlation and regression analyses reported below.

## Results

Descriptive statistics for the combined 5 years of the WWEP are presented in Table 3. Words that received the highest and lowest ratings on both variables for the 5 years are displayed in Tables 4 and 5. Below, we report analyses to address the four main goals of this project.

**Table 3:** Descriptive Statistics from the 5 years of the WWEP

Variable	*M*	*Mdn*	*SD*	*Min*	*Max*
AoA	5.00	5.20	1.07	2.06	6.75
Fam	5.91	6.27	0.94	2.55	6.93

*Note*. M = mean, Mdn = median, SD = standard deviation, Min = minimum, Max = maximum

**Table 4:** Words with highest and lowest age-of-acquisition ratings from the WWEP

Items	*M*	Length	SUBTLWF
Earliest acquired words			
Alphabet	2.06	8	2.490
Thumb	2.12	5	11.820
Jump	2.20	4	69.820
Elbow	2.24	5	6.140
Yesterday	2.39	9	96.760
Latest acquired words			
Iconoclast	6.68	10	0.020
Equanimity	6.75	10	0.600
Ergonomic	6.72	9	0.100
Theocracy	6.70	9	0.040
Listicle	6.89	8	---

*Note*. M = mean. SUBTLWF is the subtitle frequency from Brysbaert and New (2009)

**Table 5:** Words with highest and lowest familiarity ratings from the WWEP

Items	M	Length	SUBTLWF
Lowest familiarity words			
Listicle	2.55	8	---
Iconoclast	2.70	10	.020
Scrag	2.81	5	.040
Equanimity	2.89	10	.060
Zoodle	2.96	6	---
Highest familiarity words			
Yesterday	6.93	9	96.760
University	6.90	10	23.590
Body	6.88	4	195.530
Been	6.88	4	1736.730
Remember	6.88	8	542.470

*Note*. M = mean. SUBTLWF is the subtitle frequency from Brysbaert and New (2009)

### Assessing experience-related lexical change

Lexical change was first assessed by examining whether average AoA and familiarity for the entire WWEP stimulus set were changing over time. The averages of AoA and familiarity were computed across the 499 items for each of the ten semesters of data collection. Two regression analyses were computed using the semester as the predictor variable and average AoA or familiarity as the dependent variable. The averages for these variables across the ten semesters are displayed in Table 6. The semester was not a significant predictor of the average ratings for AoA (*b* = –.007, *t(*8) = –.977, *p* =.357) or for Familiarity (*b* =.006, *t*(8) =.796, *p* =.449), suggesting that average AoA and familiarity ratings for these combined set of 499 words have been stable over the 5 years of data collection for the WWEP.

**Table 6:** Average AoA and familiarity by semester for all words in the WWEP

Semester	AoA	Familiarity
1	5.06	5.85
2	4.97	5.93
3	5.02	5.93
4	5.02	5.89
5	4.95	6.00
6	5.09	5.83
7	4.97	5.81
8	5.05	5.97
9	4.86	5.90
10	5.00	6.01

**Fig. 1:**
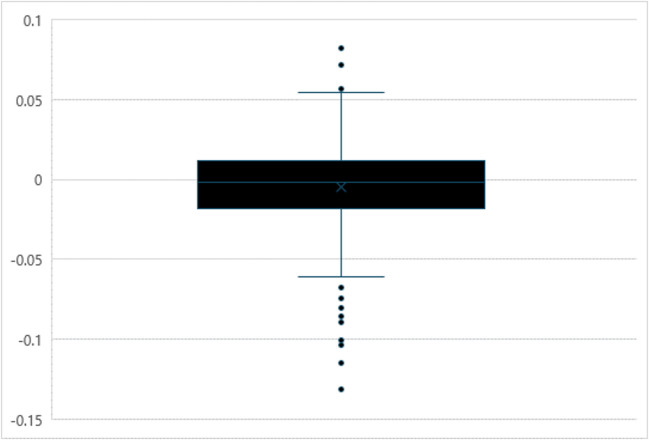
Boxplot of regression slopes used to assess lexical change in AoA over 5 years of the WWEP

**Fig. 2:**
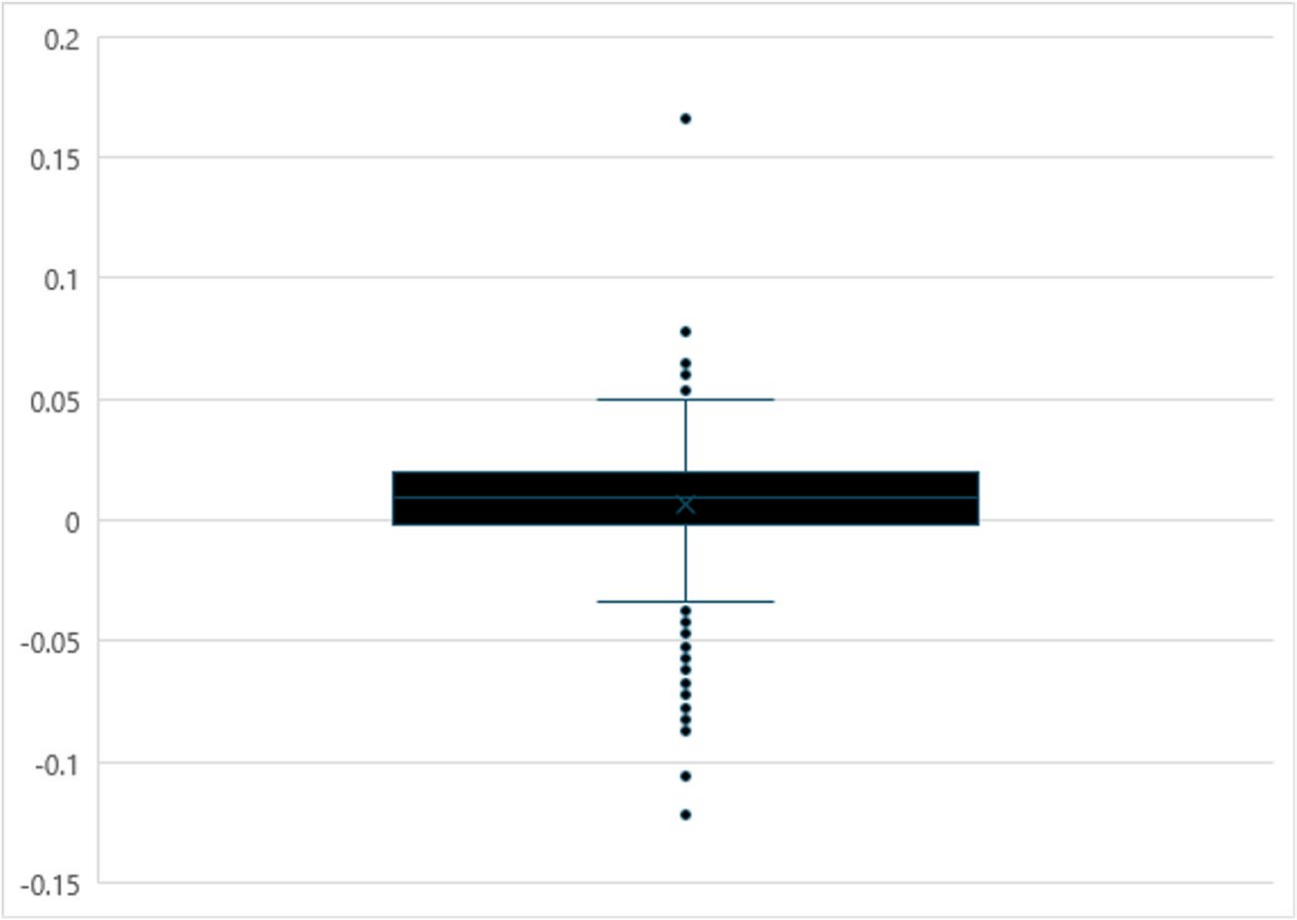
Boxplot of regression slopes used to assess lexical change in familiarity over 5 years of the WWEP

The next set of analyses examined lexical change for the individual words contained in the WWEP over a 5-year period to identify specific words that underwent lexical change. For every word, the slope of a regression line was calculated using the average rating for each semester as the dependent variable and the semester as the predictor variable. The five-number summary was then computed for the slopes for each variable, and the interquartile range (IQR) was calculated. These descriptive statistics are displayed in Table 7. The distributions of the slopes of each variable are visualized using boxplots in Figs. 1 and 2. Lexical change was quantified as outliers on both the upper and lower ends of each variable’s distribution using Tukey’s 1.5*IQR rule.

**Table 7:** Descriptive statistics for the slopes measuring lexical change for AoA and familiarity

Measure	AoA slope	Familiarity slope
Min	– 0.131	– 0.122
Q1	– 0.018	– 0.002
Median	– 0.002	0.010
Q3	0.012	0.020
Max	0.082	0.166
IQR	0.030	0.022
1.5*IQR	0.045	0.032
Lower bound	– 0.063	– 0.034
Upper bound	0.056	0.052

*Note*. Min = minimum, Q1= first quartile, Q3 = third quartile, Max = maximum, IQR = interquartile range

#### AoA lexical change

A total of 16 words were identified as being earlier acquired over the course of the 5 years of the WWEP. Intuitively, the three words undergoing the most lexical change in terms of becoming earlier acquired are words associated with technology (e.g., smartphone, meme, emoji). On the other hand, only four words were identified as being later acquired over the 5 years of the WWEP. The top one, camcorder, is again associated with changes in technology usage over time. These words are displayed in Table 8. Independent-samples *t* tests were utilized to further explore the two lexical change conditions relative to the words with consistent AoA over time. For both analyses, the average trajectory slope was significantly different for the AoA changed words relative to the AoA consistent words. These comparisons produced a large effect size for both the words with an upward AoA trajectory compared to the AoA consistent words, *t*(3.155) = – 11.096, *p* <.001, *d* = – 3.224, 95% CI [– 0.089, – 0.050] and the words with a downward AoA trajectory compared to AoA consistent words *t*(16.334) = 18.67, *p* <.001, *d* = 4.19, 95% CI [0.080, 0.100].

**Table 8:** AoA items undergoing lexical change

Becoming later acquired: Camcorder (.082), Bellhop (.071), Eyeglass (.057), Monopoly (.056)
Becoming earlier acquired: Smartphone (–.131), Meme (–.115), Emoji (–.113), Minion (–.112), Photobomb (–.104), Vlog (–.100), Joggers (–.098), Leggings (–.089), Drone (–.086), Glamping (–.085), Lit (–.085), Gluten (–.081), Swag (–.074), Tweet (–.072), Kale (-.068), Flex (-.067)

*Note*. The numbers in parentheses represent the slope of the regression line used to assess the trajectory of lexical change for each item

#### Familiarity lexical change

A large number of words (35) were identified as becoming less familiar over the course of 5 years. Unlike with AoA, the words undergoing the most lexical change in familiarity are not specific to technology and represent a variety of word types and meanings (e.g., eulogize, tofurkey, iconoclast, technicolor). A total of ten words were identified as becoming more familiar over time. These also represented a variety of word meanings (e.g., feral, glamping, blockchain, wicket). These words are displayed in Table 9. Independent-samples *t* tests were again employed to further explore the lexical change conditions. Similar to AoA, the comparisons were significant with large effect sizes for both the words with an upward familiarity trajectory compared to the consistent familiarity words, *t*(9.098) = – 6.152, *p* <.001, *d* = – 3.810, 95% CI [– 0.089, – 0.041] and the words with a downward familiarity trajectory compared to consistent familiarity words *t*(37.664) = 19.146, *p* <.001, *d* = 3.96, 95% CI [0.059, 0.074].

**Table 9:** Familiarity lexical change from the WWEP

Becoming less familiar: Eulogize (–.122), Tofurkey (–.106), Iconoclast (–.087), Technicolor (–.082), Baccalaureate (–.078), Killjoy (–.072), Ascribe (–.070), Ragtag (–.069), Schism (–.068), Oscillation (–.067), Theocracy (–.062), Telethon (–.059), Osteoporosis (–.057), Nanotechnology (–.057), Stockman (–.055), Portraiture (–.054), Brushfire (–.053), Syncopate (–.051), Bellhop (–.051), Scoutmaster (–.051), Cowhand (–.047), Jeggings (–.046), Grocer (–.046), Multiplex (–.043), Boggle (–.042), Highkey (–.042), Equanimity (–.041), Java (–.040), Hallmark (–.040), Autocrat (–.040), Airmen (–.039), Stableman (–.038), Procurement (–.037), Murk (–.035), Shoeshine (–.035)
Becoming more familiar: Haptic (.053), Skort (.054), Immaculate (.060), Dune (.060), Vendetta (.062), Athleisure (.065), Wicket (.067), Glamping (.078), Blockchain (.082), Feral (.166)

*Note*. The numbers in parentheses represent the slope of the regression line used to assess the trajectory of lexical change for each item

### Relationship between variables

**Fig. 3:**
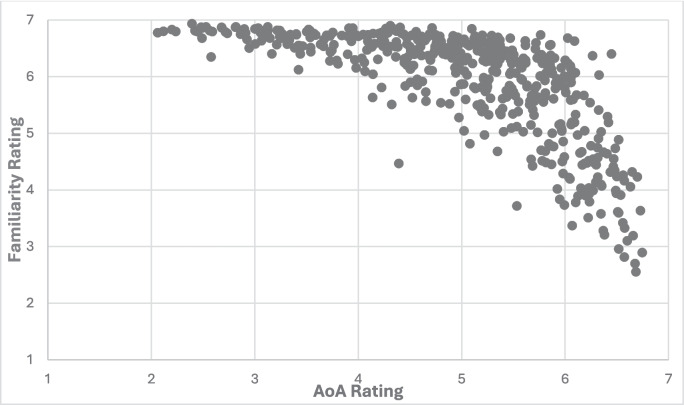
Scatterplot illustrating the correlation between average AoA ratings and average familiarity ratings for the 5 years of the WWEP. *Note*. Each point represents the AoA and familiarity ratings for an item averaged across the ten semesters of the WWEP

The relationship between AoA and familiarity across the 5 years of WWEP was explored. By-item averages were calculated across all ten semesters for each variable. A Pearson correlation was then calculated. It showed a strong, negative correlation between the two variables (*r* = –.690, *p* <.001). The size of this correlation is comparable to others reported in the literature between AoA and familiarity or subjective frequency (see Table 1). Figure 3 provides a scatterplot demonstrating the relationship between these two variables.

For the next set of analyses, Pearson correlations between AoA and familiarity were calculated for each of the 10 semesters individually. These correlations are shown in Table 10. A regression analysis was then computed using semester as the predictor variable and the correlation between AoA and familiarity as the dependent variable. This analysis demonstrated that semester was a significant predictor of the correlation between variables (*b* = –.005, *t*(8) = – 3.79, *p* =.005). The negative relationship between AoA and familiarity strengthened for the WWEP words across the ten semesters of data collection.

**Table 10:** Correlations between AoA and familiarity by semester for all words in the WWEP

Semester	Correlation
1.	– 0.634
2	– 0.655
3	– 0.669
4	– 0.665
5	– 0.665
6	– 0.698
7	– 0.663
8	– 0.680
9	– 0.700
10	– 0.689

### Interrater reliability

**Fig. 4:**
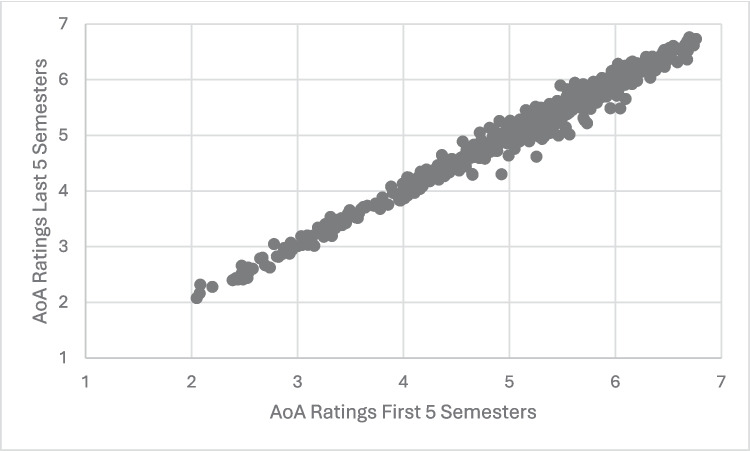
Scatterplot illustrating the correlation between average AoA ratings for the first five semesters vs. the last five semesters of the WWEP. *Note.* Each point represents the AoA ratings for an item averaged for the first 5 semesters and the last 5 semesters of the WWEP

**Fig. 5:**
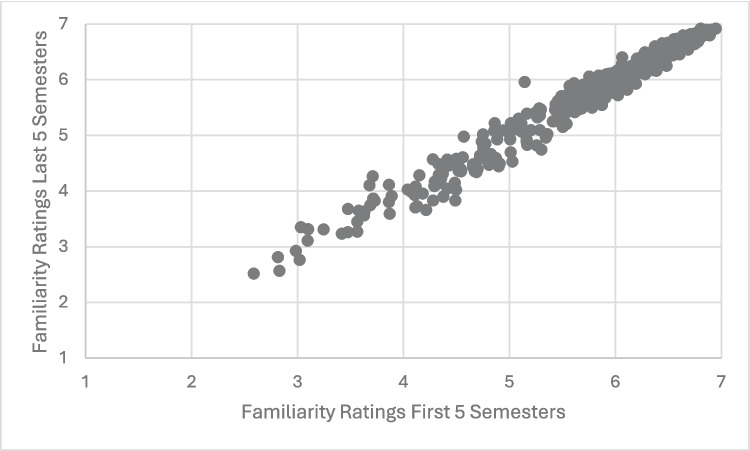
Scatterplot illustrating the correlation between average familiarity ratings for the first five semesters vs. the last five semesters of the WWEP. *Note*. Each point represents the Familiarity ratings for an item averaged for the first five semesters and the last five semesters of the WWEP

The next set of analyses addressed the interrater reliability of both AoA and familiarity over the 5 years of WWEP data collection. Specifically, the ten semesters were split in half and an average of the first five and last five semesters was computed for both familiarity and AoA. These averages were then used to calculate the by-item correlations for the two variables. Both variables demonstrated very strong correlations across the ten semesters (AoA: *r* =.991, *p* <.001; Familiarity: *r* =.988, *p* <.001). Scatterplots demonstrating these correlations are shown in Figs. 4 and 5. While both correlations are considered strong according to Cohen’s (1988) effect size guidelines for correlations (both are above* r* =.500), a Fisher r-to-z analysis demonstrated that the interrater reliability of the AoA variable was significantly higher than the interrater reliability of the Familiarity variable (*z* = 2.28, *p* <.025).

Fisher r-to-z analyses were also used to compare the correlations of the variables with themselves to the absolute value of the correlation between AoA and familiarity for the full ten semesters. The correlation of AoA with itself was significantly stronger than the correlation of AoA and familiarity (*z* = 29.16, *p* <.001). Likewise, the correlation of familiarity with itself was significantly stronger than the correlation of AoA and familiarity (*z* = 26.88, *p* <.001).

### Relationship to word recognition time from the English Lexicon Project (Balota et al., 2007)

In order to explore the relationship between the WWEP ratings of AoA and familiarity and word recognition, lexical decision and word naming reaction times were extracted from the English Lexicon Project. A total of 446 words from the WWEP were also contained in the ELP. Pearson correlations were calculated between the ratings and word recognition performance for each of the ten semesters. These correlations are displayed in Table 11.

**Table 11:** Correlations of familiarity and AoA with English Lexicon Project (ELP) recognition times

Semester	Familiarity		AoA	
	LDT	Naming	LDT	Naming
1	– 0.550	– 0.486	0.584	0.510
2	– 0.543	– 0.485	0.575	0.505
3	– 0.543	– 0.481	0.585	0.507
4	– 0.532	– 0.459	0.586	0.508
5	– 0.531	– 0.459	0.585	0.506
6	– 0.555	– 0.500	0.591	0.520
7	– 0.541	– 0.481	0.578	0.508
8	– 0.544	– 0.496	0.586	0.511
9	– 0.547	– 0.480	0.586	0.513
10	– 0.539	– 0.485	0.576	0.491

*Note*. Word recognition times are taken from the ELP ( Balota et al., 2007)

Regression analyses were computed to assess whether the correlations with ELP word recognition times were weakening over time. Contrary to predictions, there was no evidence that the relationship between AoA or Familiarity and ELP times was changing over time (all *t*-values < 1). For both AoA and familiarity, the correlations with LDT were significantly stronger than the correlations with word naming time (AoA: *t*(9) = 52.55, *p* <.001; Familiarity: *t*(9) = – 22.93, *p* <.001).

## Discussion

The WWEP tracked potential changes in the lexical experience of U.S. college undergraduates for a common set of English words over the course of 5 years (Fall 2019 to Spring 2024). Below we discuss the findings and their implications related to the project’s four main goals: 1) assess experience-related lexical change; 2) explore the relationship of rated familiarity and AoA over time; 3) explore and compare each variable’s interrater reliability; and 4) determine if the applicability of the word recognition times from the ELP are decreasing due to changes in young adults’ collective lexical experience.

Our first goal for the WWEP was to explore lexical change over the course of 5 years. As discussed by Beckner et al. (2009), patterns of language use among individuals in a society affect how features of the language are first acquired, are organized, and change over time. Language is a complex adaptive system that is shaped by patterns of language use along with other general cognitive processes (e.g., categorization, attention). At the heart of this theoretical approach is the idea that the interactions of speakers and their respective language experiences drive changes in language over time. Language change can thus be thought of as a cultural evolutionary process (Christiansen & Chater, 2008) involving both replication and selection. Words (along with other aspects of language) are replicators in this process. Speakers of a language introduce variation into the replication of words due to interactions with their environment, leading to selection differences for words. Beckner et al. (2009) note the challenge associated with measuring lexical change at the community level. The WWEP aimed to address this challenge and develop a mechanism for tracking lexical change within an undergraduate college community.

In service of our first goal, and consistent with our expectations, we identified a number of words that are undergoing lexical change in their overall familiarity and/or the age at which undergraduate college students report acquiring them. Notably, we did not observe changes in the average AoA or familiarity to the entire list of words over time. Most words in the WWEP had slopes that were close to zero, illustrating the consistency with which these words are rated on familiarity and AoA by separate groups of undergraduate raters over the course of 5 years. It is therefore not the case that college students have changed HOW they use the rating scales as a whole over the past 5 years. The words that were identified as undergoing lexical change, which are displayed in Tables 8 and 9, demonstrate that changes in experiences with words can be tracked at the level of the individual lexical items over time.

Interestingly, only two words were identified as undergoing lexical change for both the age at which they are reported to first be learned and how familiar they are. The compound word “bellhop” was identified as becoming later acquired and less familiar over the past 5 years. Conversely, the word “glamping”, which is a blend of *glamorous* and *camping*, was identified as becoming earlier acquired and more familiar. The remaining 61 words that were identified with our approach are unique exemplars of experience-based lexical change. When examining the specific items undergoing lexical change, it is notable that the top three words that were identified as becoming earlier acquired are related to technology, illustrating the relationship between changes in society and changes in word usage over time. This supports the point raised by Beckner et al. (2009) that *“changes in lifestyle lead to the rise and fall of words and constructions associated with those lifestyles”* (page 9). Changes in uses and types of technology over time lead to lexical change. While the current methods have identified words undergoing lexical change, it should be noted that the current ratings do not take into consideration changes in the senses/meanings of words that may be occurring. Future studies could specifically probe meaning changes over a certain time period by asking participants to provide an associated word, for example.

The second goal of the WWEP was to explore the relationship between AoA and familiarity. As mentioned in the Introduction, these two variables both assess individual experience with words and have been linked to both lexical and semantic levels of processing in the mental lexicon (Juhasz et al., 2015). In support of this relationship, we observed a strong, negative correlation between the two variables in the current study (*r* = – 0.690). Consistent with our expectations, the magnitude of this correlation is remarkably similar to those reported in the past studies, as displayed in Table 1. There has been some discussion in the literature about how the difference in instructions for general “familiarity” ratings compared to “subjective frequency” (e.g., Brysbaert & Cortese, 2011) may cause raters to differentially recruit the use of semantic information. This may lead to differences in the observed effects of these variables on word recognition. However, Table 1 shows that the correlation between these two variables is stable whether the instructions for the familiarity variable stressed overall familiarity with the words (as was the case in the current study) or focused on subjective frequency of occurrence. In both cases, words that are rated as being acquired earlier in life are also rated as being more familiar. Contrary to our initial expectations, the relationship between these variables strengthened over the 5-year period. According to the LQH (e.g., Perfetti, 2017), words that are high in lexical quality in the mental lexicon have well-specified representations and are therefore faster to recognize. Highly familiar, early acquired words are therefore likely to have stronger lexical quality and thus be easier for an individual to recognize during reading. Future research could directly compare ratings of overall familiarity and subjective frequency for the same word sets to see if there are differences in their interrater reliability over time.

While AoA and familiarity are clearly related, the current study also provides supportive evidence that they are not measuring the same underlying construct. The correlation of AoA with itself and the correlation of familiarity with itself over the ten semesters were both significantly stronger than the correlation between AoA and familiarity. With respect to the third goal of this project, both AoA and familiarity demonstrated high interrater reliability over the course of the 5 years of data collection, consistent with expectations. When directly compared, however, the correlation of the first five semesters and last five semesters of AoA was significantly stronger than that for familiarity. As mentioned in the Introduction, there has been debate in the literature as to whether college students’ ratings of AoA can provide a useful insight into word recognition processes (e.g., Wikse Barrow et al., 2019). In addition to the high correlation we observed, average AoA for the entire set of WWEP words did not significantly change with time. While the current study does not speak to the construct validity of AoA ratings, it does provide strong support that the information that students are using to make their AoA ratings is stable over time and there is a high degree of consistency in such ratings.

Given that average AoA and familiarity were relatively consistent over the course of the 5 years of data collection, it is perhaps unsurprising that the correlation between these ratings and the word recognition times for the 446 words contained in the ELP was likewise stable over time. This is contrary to our initial expectations. The ELP was published in 2007 (Balota et al., 2007), with data collection having occurred at U.S. colleges and universities prior to publication. We had thus expected the relationship between experience-based ratings, such as familiarity and AoA, and the ELP word recognition times to weaken over time. In contrast, the correlations with both LDT and naming time did not significantly decrease across the ten semesters of data collection. The ELP thus remains a viable resource for exploring how lexical experience impacts word recognition times. This finding should be beneficial for researchers who utilize the ELP to select items, test novel hypotheses, and replicate their own individually collected laboratory-based word recognition tasks.

There are limitations to this study that should be addressed. First, due to constraints on the availability of the participant pool, the number of raters differed each semester. In addition, participants could choose to skip rating words within the questionnaire. Ideally, the sample size for each semester would be equivalent and future studies should aim to include a more stable number of participants per semester. In order to address this limitation, the number of observations per item is included for each of the ten semesters of the WWEP in the supplemental information so that interested researchers can take this variation into consideration. Second, while the focus on one college in the Northeast of the U.S. reduces potential variations in geographical region, educational status and age ranges of participants, it also limits the generalizability of the resulting observed lexical change to other language-user groups. It would be interesting to conduct a similar multi-year study with individuals across the U.S. to explore region-specific changes that may be occurring. Future studies could specifically focus on bilingual individuals or those from different educational backgrounds to enhance generalizability. Likewise, exploring these changes in different age groups (e.g., children, adolescents, younger adults, and older adults) could provide a more accurate picture of collective lexical change for different language-user groups. In addition, the word set was restricted to 499 words that were mostly randomly selected from the English Lexicon Project. Future studies could expand this list to include additional words selected from targeted sources (e.g. media, technology, scientific vocabulary) in order to further explore lexical change in specific vocabulary domains.

While 5 years of data collection was the plan for the initial time period of the WWEP, it would also be beneficial to expand data collection to a longer time period. The current time period identified 63 individual words that are undergoing experience-related lexical change. However, on average, the ratings for the full set of words on both AoA and familiarity remained stable. Expanding the collection of data to 10 years or longer may support the identification of additional lexical items undergoing lexical change and reduce the chance that the current findings are due to random variation.

In conclusion, this is the first study, to our knowledge, that has been able to track experience-related change for individual lexical items. Language change can be thought of as a cultural evolutionary process (Christiansen & Chater, 2008) that has been difficult to study experimentally in the past due to the timescale associated. The WWEP provided a viable mechanism to measure lexical change over the course of 5 years to English words with one particular undergraduate college population. The project also provides additional evidence that ratings of AoA and familiarity by college students are reliable over time, thus further supporting the use of these subjectively rated variables in word recognition research.

## Data Availability

Ratings and number of observations for all words contained in the Wesleyan Word Experience Project Database. The database is available here: https://osf.io/tqckx/?view_only=6478df9285c24b329d9f1330089397cc This project was not preregistered.
